# Recent applications of magnetic iron oxide nanoparticles for cerebral blood volume quantification in functional magnetic resonance imaging

**DOI:** 10.1002/ibra.70004

**Published:** 2025-10-11

**Authors:** Giorgio Capuzzello, Antonella Antonelli, Rosaria Rinaldi, Riccardo Di Corato

**Affiliations:** ^1^ Department of Mathematics and Physics “Ennio De Giorgi” University of Salento Via Arnesano Lecce Italy; ^2^ Department of Biomolecular Sciences University of Urbino Carlo Bo Via Cà Le Suore Urbino Italy; ^3^ Institute for Microelectronics and Microsystems (IMM) Consiglio Nazionale delle Ricerche (CNR) Via Monteroni Lecce Italy

**Keywords:** blood oxygenation level dependent, cerebral blood volume, contrast agents, functional magnetic resonance imaging, iron oxide nanoparticles

## Abstract

The use of iron oxide nanoparticles (IONPs) in magnetic resonance imaging (MRI) and the study of brain functions using MRI have been continuously evolving for more than 30 years. This contribution aims to explore the recent applications of magnetic IONPs in the study of the brain through functional MRI (fMRI), particularly focusing on their use in a specific parameter, that is, the cerebral blood volume (CBV), whose measurements are valued for their higher sensitivity and spatial specificity with respect to blood oxygen level‐dependent (BOLD) fMRI analyses. This study will summarize the basis of the fMRI technique, explaining the types of experiments commonly conducted, the parameters involved, and discussing the main techniques that exploit magnetic nanoparticles and other materials as contrast agents, along with their potential applications in the imaging field. CBV measurements will be explained in general theoretical terms and compared with other methods. The key elements of magnetic nanoparticle production, including the most commonly used synthesis procedures and coating options, will be reported. Finally, the discussion will focus on how CBV‐weighted (CBVw) images acquired using IONPs are currently being utilized in research.

## Graphical abstract

This study explores the recent advances in the use of iron oxide nanoparticles (IONPs) in functional MRI (fMRI) for studying brain functions, specifically cerebral blood volume (CBV) measurements. It covers fMRI techniques, magnetic nanoparticle synthesis, coating methods, and their applications in CBV imaging, in comparison to other contrast agents in brain research.

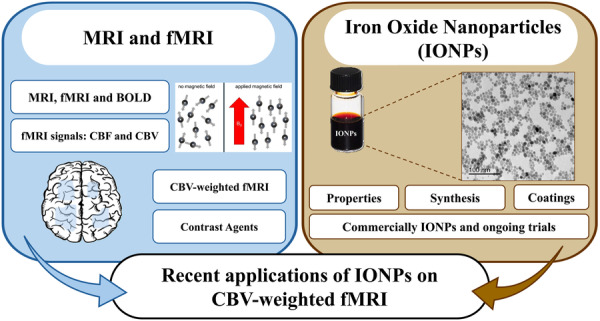

## 1 INTRODUCTION

Functional magnetic resonance imaging (fMRI) was introduced in the early 1990s^1^, ^2^ with the aim of highlighting changes in brain areas related to neuronal functionality in real‐time in a noninvasive way. Several acquisition methods have been implemented since the first studies, and they are continuously updated to obtain increasingly high‐resolution and well function‐related images, as well as to extend the use of fMRI as a standard for medical and research applications.^3^, ^4^, ^5^, ^6^

**Figure 1:**
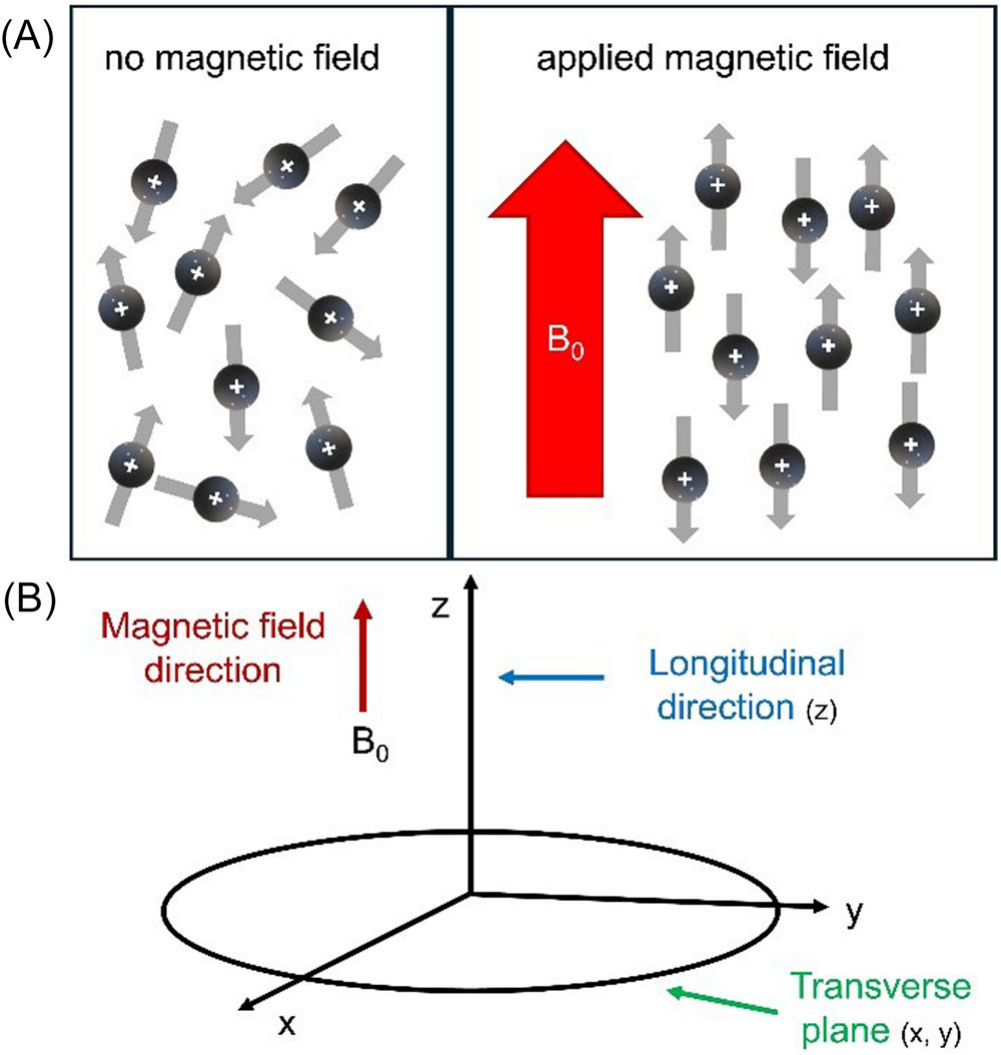
The schematic vision of hydrogen proton alignment under the magnetic field B_0_ and the coordinate system. (A) Before the field application, the hydrogen protons were oriented randomly (left panel), then their magnetic moments were aligned following the direction of the magnetic field (right panel). (B) The *z* axis represents the longitudinal direction, usually aligned with the main magnetic field. The perpendicular plane is called the transverse plane (x, y). [Color figure can be viewed at wileyonlinelibrary.com]

Magnetic resonance imaging (MRI) is based on the application of a magnetic field that causes an alignment of the hydrogen atoms nuclei magnetic moments. The atomic nuclei will spin around their own axes at a specific frequency called Larmor's frequency. By applying a radiofrequency (RF) pulse with the same frequency of Larmor's frequency, the nuclei will acquire energy that brings them into an unstable state. After the RF pulse is interrupted, the realignment of the magnetic moments of the hydrogen nuclei (relaxation) is captured and used as a signal for imaging. Relaxation times are the parameters that need to be considered for image generation: the longitudinal relaxation time (T1) indicates the time needed for the decay of the longitudinal magnetization after the RF pulse, whereas the transversal relaxation time (T2) consists of the time needed for the decay of the transversal magnetization after the RF pulse (Figure 1). The soft tissue contrast provided by MRI arises principally from differences in the intrinsic relaxation properties, T1 and T2; despite the intricate relationships linking tissue microstructure and the longitudinal and transverse relaxation times remaining to be tightly established, quantitative measurements of these parameters can be informative of disease‐related tissue change. Iron oxide particles are excellent contrast agents for molecular and cellular MRI because of their large effect on water proton transverse relaxation.^7^, ^8^ In this way, by targeting molecules that contain hydrogen atoms, it is possible to visualize and distinguish different tissues of the body, thus allowing the detection of the differences between physiological and atypical conditions in the same tissue or organ.^9^, ^10^

With fMRI, changes in blood flow and oxygenation derived from the neuronal activity are measured, obtaining whole‐brain functional mapping in both animals and humans.^11^ The first fMRI method is called blood oxygen level‐dependent (BOLD) fMRI (BOLD‐fMRI)^12^ because it provides an indirect measure of neuronal activity by exploiting deoxy‐hemoglobin as an endogenous contrast agent, achieving a spatial resolution of a few millimeters.^10^, ^11^, ^13^ For this reason, brain anatomy and physiology are useful for comprehending the technique, starting with neuronal activity at the neurotransmitter level and extending to the activation of the different brain areas involved in various functions.^14^

This review will introduce some general aspects of the fMRI technique and acquisition methods, starting with BOLD‐fMRI and highlighting some fundamental parameters for brain mapping, including cerebral blood flow (CBF) and cerebral blood volume (CBV), mentioning the relationship between them and how they are used in fMRI. It will introduce iron oxide nanoparticles (IONPs), which are widely used in MRI and fMRI, summarizing their properties, the main synthesis methods and their role in MRI as contrast agents, comparing with other contrast agents like gadolinium‐based ones. The discussion in the final section will be focused on the recent advances in fMRI studies that exploit IONPs for the CBV measurements, underlining the main advantage of CBV fMRI with IONPs compared to BOLD‐fMRI, which lies in its greater sensitivity and spatial specificity.^15^, ^16^

## 2 FMRI

### 2.1 fMRI and BOLD‐fMRI basis

The fMRI aims to investigate which brain areas are involved in functional processes and how the various regions interact and cooperate with each other to perform a specific brain function, by exploiting changes in cerebral haemodynamics in response to neuronal activity.^17^, ^18^, ^19^ Several images are acquired in a single experiment, capturing individual brain areas or the entire brain (the smallest element is called a voxel) following an input, such as sensory stimulation, a cognitive task, or spontaneous brain activity.^17^ In this way, it is possible to establish a connection between brain function and brain regions, and to apply this in research, such as studying the alterations in activation patterns after brain damage, like stroke, or also evaluating pharmacological treatments.^20^

BOLD contrast characterizes the majority of the fMRI acquisitions.^18^ Deoxy‐hemoglobin is paramagnetic and present in red blood cells. This means that it can be used as an endogenous contrast agent because its concentration changes when intracellular and extracellular events occur during a group of neuron activation, and due to its paramagnetic property, it can affect magnetic field gradients around blood vessels.^17^, ^21^, ^22^ This implies that neuron activity is reflected in metabolic and haemodynamic changes, leading to local deoxy‐hemoglobin concentration variations. As a result, capillaries and veins can cause alterations in magnetic fields around them, which can be detected with T2*‐weighted MRI to measure parameters like CBF and CBV.^17^, ^21^, ^23^, ^24^

Among the metabolic changes, it is known that neuronal activity is a process that needs energy consumption for the release and the uptake of the neurotransmitters involved in synaptic transmission. Energy consumption means adenosine triphosphate (ATP) consumption, which is an oxygen and glucose‐dependent intracellular process. Both oxygen and glucose are blood‐delivered and the process causes several changes in blood molecule concentration, blood afflux for neuron activity support, and blood vessel size. In other words, when there is a local neuron activation, the oxygen and glucose consumption trigger the delivery of oxygenated blood to the functioning site coupled with vasodilation. For this reason, it is possible to relate these events with parameters like CBF and CBV (increased together with deoxy‐hemoglobin), and this explains why they represent an indirect measure of neuronal and brain activity.^21^, ^25^, ^26^, ^27^

### 2.2 fMRI studies and applications

There are two main types of fMRI experiments: task‐based fMRI and resting‐state fMRI. Also, there is pharmacological fMRI (phMRI) for the evaluation of pharmacological treatments.

#### 2.2.1 Task‐based fMRI

**Figure 2:**
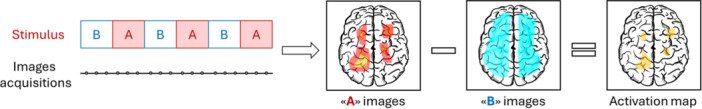
Schematic representation of task‐based fMRI acquisitions. Several images are acquired while the subject performs a given task under two conditions: A represents stimulus “on,” while B represents stimulus “off.” Comparing images acquired during conditions A and B, it is possible to highlight significant signal differences in each voxel. The stimulus is repeated multiple times to enhance the signal and average out the noise, thereby allowing for a more accurate statistical analysis. [Color figure can be viewed at wileyonlinelibrary.com]

In task‐based fMRI, several images are acquired in a short time while the studied subject performs a given task, which could be a motor, sensory, or cognitive task, or a response to external stimuli, to stimulate the brain activity. In this way, it is possible to compare images acquired before, during, and after the task, mapping the brain regions that undergo changes during the neuronal activity by correlating the signal time course in each voxel to the expected modeled evoked signal.^19^, ^23^, ^26^, ^28^, ^29^ Tasks and control conditions are repeated multiple times, providing accurate statistical data and maximizing the technique's sensitivity^24^ (Figure 2).

An example of this approach can be found in a recent study by Pan et al., who studied aberrant brain network topology in young individuals with depression and/or anxiety disorders and a familiar history of bipolar disorders (BD). The study intends to identify new biomarkers using a task‐based BOLD‐fMRI during an emotional continuous performance task. The fMRI acquisitions revealed distinct brain connectivity abnormalities in the BD risk‐group with respect to control group, suggesting how altered brain‐network architecture represents a neurobiological marker for familiar BD risk in symptomatic adolescents.^30^

#### 2.2.2 Resting‐state fMRI

Instead, in resting‐state fMRI, the spatial functional correlations of the BOLD signal are studied without stimulation or task‐execution.^19^ The theory is that different brain regions' fluctuations over time are temporally synchronized and organized as nodes in networks.^31^ The acquisition methods are similar to task‐based fMRI.^26^, ^32^ He et al. used resting‐state BOLD‐fMRI for the comparison of brain functions in major depressive disorders (MDD) and social anxiety disorder (SAD), collecting data from patients affected by these disorders and healthy controls and making a statistical comparison between them. Among the acquisition methods, they collected gradient echo‐planar imaging sequence for resting‐state functional BOLD images, discovering that patients showed common and distinct aberrant brain function patterns at regional and network levels.^33^

As seen from the previous examples, the use of fMRI in neurological disorders research is just one of the possible applications of the technique in clinical research in humans.^34^, ^35^, ^36^

#### 2.2.3 phMRI

Another method used for the activation of neuronal function is the phMRI, in which the neuronal activation occurs due to pharmacological stimulation. This means that the haemodynamic changes used for the study of cerebral activity are evoked by a pharmacological agent, enabling the evaluation of new therapies.^19^, ^37^, ^38^ The dose–response effects and the pharmacokinetics of the administered drug should always be considered, particularly regarding the systemic effects on physiological functions such as brain haemodynamic response, to avoid alterations in fMRI measures.^37^, ^39^ An example of this method in mice can be seen in Perera et al.'s work.^40^ They performed simultaneous recordings of pharmacological perturbation of brain perfusion and blood‐cerebrospinal fluid barrier (BCSFB) function after the use of vasopressin or caffeine injected intraperitoneally, using arterial spin labeling for image acquisition. They demonstrated that there are different responses to vasopressin and caffeine derived from the different physiology of the blood‐brain‐barrier (BBB) in the vessels that perfuse the cortex and the BCSFB within the choroid plexus, discovering that there is an aging‐dependent response, and that caffeine is the better candidate for BCSFB vasculature studies.^40^ The role of caffeine in BBB permeability has also been revealed by Lin et al., in their 2022 study, in which they report that caffeine consumption is responsible for alterations in CBF and oxygenation without BBB permeability alteration.^41^

### 2.3 fMRI signals: CBF and CBV

There are several parameters coupled with neuronal activity that are used in fMRI as signals. One of the primary signals commonly used is CBF, which reflects neuronal activity for its role in glucose and oxygen apport to the brain.^42^ CBF is defined as the rate of delivery of arterial blood to the capillary beds of a determined mass of tissue; for this reason, when it is used for imaging applications, it is usually expressed as blood flow delivered to a unit volume of tissue.^42^ Instead, CBV is a physiological parameter that indicates the amount of blood in brain tissue or the fraction of the tissue volume occupied by blood vessels, expressed in milliliters of blood on milliliters of tissue.^42^, ^43^ Notably, CBV is used as an indicator for early stages of cerebrovascular alterations because it can be considered as the main modulator of BOLD effect, and is useful for its interpretation.^43^, ^44^

The relationship between CBV and CBF lies in the fact that inside the brain, during neuronal activation, CBF is locally increased due to a reduction in cerebral vascular resistance, and this results in an increased CBV. Cerebral vascular resistance is based on CBF and is related to a pressure decrease in blood vessels from large arteries to large veins.^45^ This also means that in a healthy brain, an increased CBF is paired with an increased CBV as a consequence.^42^ This correlation can be disrupted in brain disease, for instance, in ischemic states.^42^, ^46^, ^47^

The central volume principle describes perfectly this relationship between CBF and CBV, introducing a parameter called mean transit time *τ*, which represents the time spent by blood or a blood component within a defined volume of a blood vessel such as a capillary.^42^, ^48^

(1) $$\tau =\frac{\mathrm{CBV}}{\mathrm{CBF}}.$$

Usually CBV value in a healthy brain is around 4%, but it could vary based on the arteries, veins or capillaries.^42^ CBF is instead considered to have a mean value of 60 mL/100 grams per min (0.01 s^−1^). Taking these main values as an example, the mean transit time of a determinate volume of blood in a determinate volume of blood vessel, following Equation 1, is approximately 4 s.^42^

### 2.4 Contrast agents and CBV measurements

Among the imaging techniques developed for the measurement of CBV, the most common ones rely on the use of a contrast agent that separates signals coming from intravascular and extravascular compartments of the tissues (CBV refers to the intravascular space).^43^ Contrast agent techniques could be divided into two main categories: bolus tracking techniques, in which an intravascular contrast agent is used to modify the magnetic susceptibility of blood inside vessels; arterial spin labeling, which consist in tagging magnetically the arterial blood before its diffusion inside the tissue to measure the amount of it that has been delivered inside the tissue. The first method is used for CBV quantification and the second method is mainly used for CBF.^42^, ^49^, ^50^

#### 2.4.1 Gadolinium diethylenetriamine pentaacetate (Gd‐DTPA)

The first approach for measuring CBV in fMRI was performed by injecting paramagnetic gadolinium‐chelate contrast agent in a task‐based human study.^49^, ^51^ Gadolinium‐based agents have a high magnetic moment and good stability, advantages derived from the absence of electrons bounded.^52^ The most common gadolinium‐based agent is Gd‐DTPA, commercially available as Vasovist®. When an external contrast agent is injected, its delivery into the brain depends on CBF and on the characteristics of the tissue.^52^ Gadolinium is a lanthanide with seven unpaired electrons that generate a strong magnetic field, promoting the relaxation of nuclear magnetic moments around them. Specifically, the effect of Gd‐DTPA is to reduce the relaxation time T1 of surrounding water molecules to have an increased signal in T1‐weighted images.^42^ The main problem with gadolinium‐based contrast agents is their short blood half‐life. This makes it a good candidate for CBF measurements that do not require a contrast agent with a prolonged lifetime in blood vessels.^42^, ^52^ Apart from general issues with gadolinium (i.e., increased risk for nephrogenic systemic fibrosis in patients with severe renal insufficiency and/or hepato‐renal syndrome, or in patients in the perioperative liver transplantation period^53^), the main limitation of CBV‐weighted (CBVw) fMRI with gadolinium‐based contrast agents is their short blood half‐life due to a fast extravasation.

#### 2.4.2 IONPs

CBV measurements require a contrast agent with a longer half‐life such as IONPs, which are used for their biocompatibility, ease and plasticity of synthesis and functionalization and for their magnetic and superparamagnetic properties (diameter <25 nm).^52^ For this purpose, the IONPs are used in small sizes to have superparamagnetic particles, usually referred to as superparamagnetic IONPs (SPIONs).^54^ In this field, different names are used for the same or similar types of nanostructure based on the diameter of nanoparticles (NPs), such as monocrystalline iron oxide nanocrystals (MION)^55^ (4–25 nm of core size)^42^ or ultrasmall SPIONs (USPIO)^56^ (4–10 nm of core size).^42^, ^52^ MION is also used as a general term to refer to all types of IONPs.^57^ Blood prolonged half‐life and biodistribution are essential for the use of the contrast agents and are influenced by both the hydrodynamic diameter and the coating of the nanostructures. The coating is responsible for the surface charge and the physiological compatibility of the NPs, involved in IONPs absorption by spleen, liver, bone marrow, and lymph nodes, as well as their bioaccumulation and elimination. The most common method for functionalizing IONPs for MRI use is the dextran coating, using both dextran^58^ and its derivatives, such as carboxymethyl dextran.^52^, ^59^ These polymer‐coated NPs usually play a fundamental role in T2 and T2* shortening and in enhancing the contrast in transverse T2‐weighted images, in a stronger way rather than gadolinium‐based paramagnetic contrast agents, and require less concentration for their use in MRI.^59^, ^60^

The comparison between magnetic NPs and gadolinium‐based contrast agents is always relevant. An interesting study was carried out in 2021, when Wei et al. compared their single‐nanometer IONPs (SNIO) with Gd‐DTPA. Their system is composed of IONPs synthesized by thermal decomposition and water‐transferred with a coating of zwitterionic dopamine sulfonate (ZDS), with a hydrodynamic diameter around 3 nm (including an iron oxide core of 1 nm). Their NPs are weakly superparamagnetic and more similar to Gd‐DTPA, suggesting their role in signal brightening in T1‐weighted MRI, and specifically producing a per‐molecule longitudinal relaxation enhancement 10 times greater. Their advantage also consists in their ability to permeate through tissues like gadolinium‐based contrast agents, in contrast to bigger IONPs penetrating the BBB after ultrasound‐induced disruption.^61^

## 3 IONPS: PROPERTIES, SYNTHESIS, AND APPLICATIONS

### 3.1 Magnetic properties of IONPs

Magnetic NPs are defined as a class of NPs with magnetic properties derived from their composition (mainly iron oxide), and with core dimensions under 100 nm.^60^ Iron oxide composites are widely spread in nature, easy to synthesize, inexpensive, biocompatible (property enhanced by the coating procedures), physically and chemically stable, and environmentally safe. For these reasons, they find application in several fields ranging from drug delivery to environmental remediation processes.^62^, ^63^ In this context, they are considered for their use as contrast agents in MRI and fMRI.

Among iron oxides present in nature, the most common are hematite (α‐Fe_2_O_3_), magnetite (Fe_3_O_4_), and maghemite (γ‐Fe_2_O_3_). The last two are predominantly used in the biomedical field for their major stability and their superparamagnetic properties.^64^

Magnetic materials are composed of magnetic dipoles generated by electron spins. Each polarized electron can be oriented parallel or antiparallel to the other surrounding electrons inside the crystal lattice. Essentially, magnetic materials are classified based on their response to the application of an external magnetic field. These can be paramagnetic, ferromagnetic, antiferromagnetic, and so forth. This depends not only on their composition and crystal lattice organization, on the nanoparticle dimensions, but also on external parameters such as temperature.^65^, ^66^ Magnetite and maghemite are both ferrimagnetic iron oxides, which exhibit strong magnetic ordering due to antiparallel alignment of magnetic moments, resulting in a significant net magnetic moment. This behavior makes them highly sensitive to external magnetic fields, which is crucial for nanomedicine applications.^58^, ^67^ Although maghemite shares the inverse spinel structure with magnetite, it is a defective spinel with cation vacancies, which can affect its magnetic anisotropy, showing a lower magnetization than magnetite. In contrast, hematite is mainly antiferromagnetic below its Néel temperature, meaning that its magnetic moments largely cancel, leading to a very weak net magnetization.^68^ Consequently, magnetite and maghemite are strongly magnetic and, under a certain threshold, show a peculiar superparamagnetic behavior, while hematite is generally weakly magnetic, finding sporadic application in the biomedical field.

In particular, superparamagnetism is the property that enables the NPs to behave as a single paramagnetic atom, with a quick response to the influence of external magnetic fields, showing no coercivity and nonrelevant residual magnetism at room temperature. Coercivity refers to the coercive field, which is the external magnetic field with opposite sign needed to bring the magnetization to zero. A paramagnetic material has more magnetic dipoles randomly oriented due to thermal agitation, and their magnetization can be saturated under the influence of an external magnetic field. As a consequence, the material exhibits high magnetic susceptibility and the consequent loss of magnetization with the external magnetic field removal. This behavior characterizes superparamagnetic NPs, and is important to note that this property occurs when the size of NPs is less than 25 nm, otherwise, at higher diameters, the ferromagnetic behavior takes over (this means that after the application of the magnetic field, there is a collective response of the magnetic dipoles that align parallel to each other). This phenomenon occurs because, inside these small NPs, at room temperature, the thermal energy of the lattice is comparable to or greater than that needed to overcome the energy barrier that blocks the dipoles of a given state/alignment. These properties are fundamental for in vivo applications such as in MRI, and in particular, the absence of coercivity that decreases with dimensions prevents NPs from aggregating. Another important aspect for preventing NPs aggregation resides in the coating of NPs, because without it they tend to aggregate to reduce the energy associated with the high surface‐volume ratio. Therefore, a coating is applied, which can be composed of organic (e.g., polymer coating) and inorganic materials (e.g., formation of core‐shell nanostructures).^62^, ^63^, ^65^, ^66^, ^69^, ^70^, ^71^, ^72^, ^73^

### 3.2 Synthesis methods

**Figure 3:**
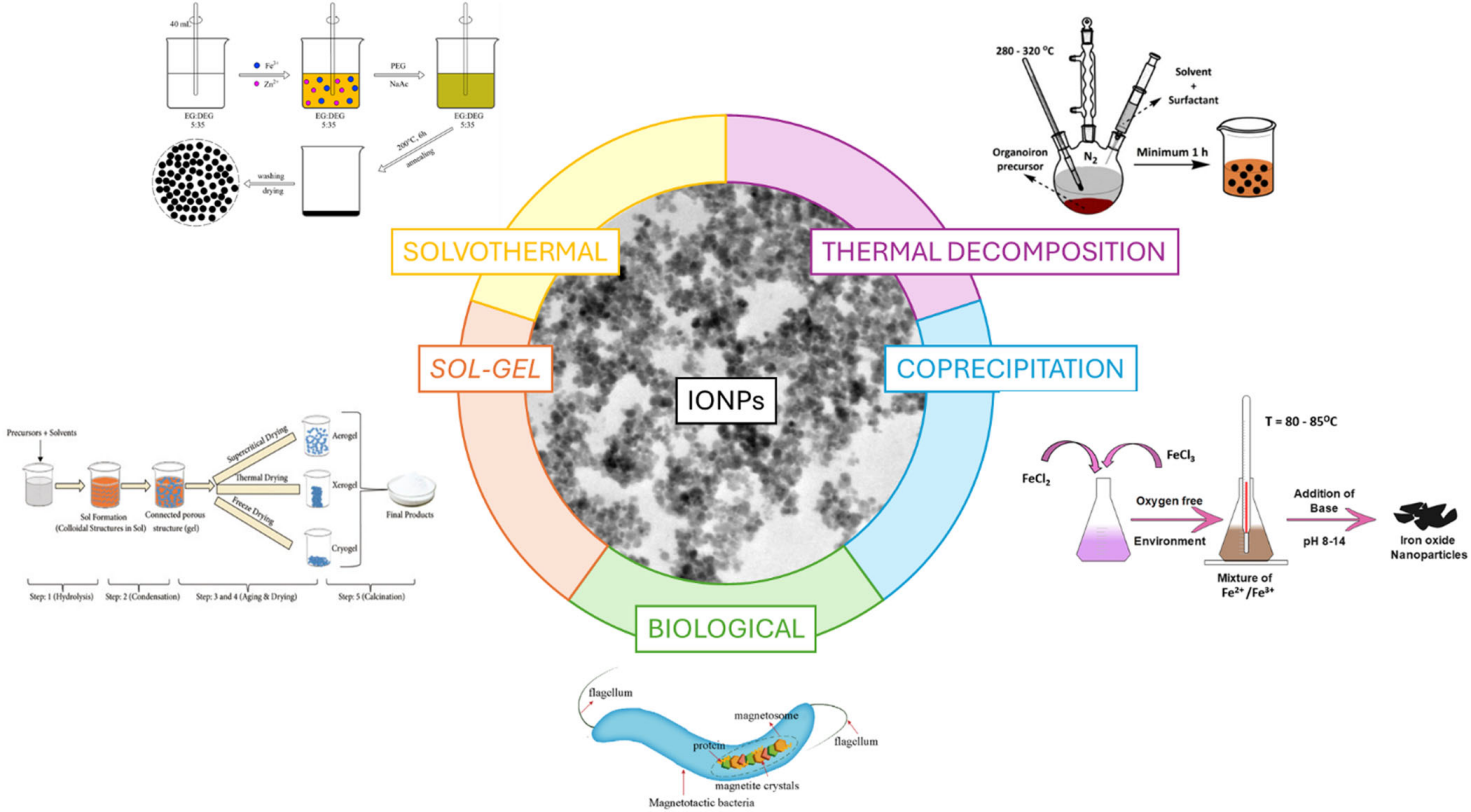
The synthesis methods of iron oxide nanoparticles (IONPs): thermal decomposition,^78^ co‐precipitation,^79^ biological,^80^ sol‐gel^81^ and solvothermal^82^ methods. Each synthetic method produces nanoparticles with a peculiar size, shape, distribution or crystallinity purity. The image reported in the figure refers to a typical co‐precipitation synthesis product. [Color figure can be viewed at wileyonlinelibrary.com]

The synthesis of superparamagnetic NPs is a complex process due to their colloidal nature.^71^ Synthesis methods for IONPs are divided into two main categories: chemical‐physical methods and biological methods (i.e., the use of bacteria^74^). Within the first category, there is a further division into aqueous and organic methods, which include the most common methods: co‐precipitation,^58^ thermal decomposition,^75^ solvothermal^76^ and sol–gel^77^ methods (Figure 3). Co‐precipitation and thermal decomposition are the preferred methods for synthesizing NPs for biomedical applications, especially in MRI. The aim of the synthesis for biomedical applications is to have high control over dimensions, shape, composition, and stability of the suspension of NPs, where iron oxides need to be monodisperse without forming aggregates or precipitates; all of these parameters have a significant influence on their magnetic properties.^63^

#### 3.2.1 Co‐precipitation

The co‐precipitation synthesis is one of the most commonly used methods because it is easy, fast and low cost; however, it allows only limited control of dimensions, shape and composition of NPs. This variability is the result of multiple factors such as the iron salts used (chlorides, perchlorates, sulfates, nitrates, etc.), Fe(II)–Fe(III) ratio, temperature, surfactants, pH value and ionic strength of the solution.^62^, ^63^, ^70^, ^83^

The synthesis involves the use of Fe(II) and Fe(III) salts in an aqueous solution and a basic environment. The molar ratio between iron salts is generally 1:2, and the reaction could be carried out at room temperature or by heating the solution. The reaction mechanism can be simplified using the reaction down below.^63^, ^65^, ^70^, ^83^

$${\mathrm{Fe}}^{2+}+2{\mathrm{Fe}}^{3+}+8{\mathrm{OH}}^{-}⇆\mathrm{Fe}{\left(\mathrm{OH}\right)}_{2}+2\mathrm{Fe}{\left(\mathrm{OH}\right)}_{3}\to {\mathrm{Fe}}_{3}O_{4}↓+4H_{2}O.$$

The nucleation step occurs at pH < 11, while the growth of the nanoparticle seeds happens at pH > 11. Thus, varying pH values and the amount of the base (usually NaOH or ammonia), spherical IONPs can be produced with different sizes.^79^ Polymer coating procedure could be performed during or after the synthesis, and provides a greater colloidal stability and increased biocompatibility of NPs in physiological conditions.^63^, ^65^, ^70^

#### 3.2.2 Thermal decomposition

One of the main limitations of the co‐precipitation method is the achievement of polydisperse NPs due to their fast formation. Other synthesis strategies have been developed for the preparation of monodisperse and highly crystalline samples, based on a nucleation step and a slow and controlled growth step. Thermal decomposition belongs to organic methods and happens at high temperature, and it is mainly used for the synthesis of semiconductor nanocrystals. The synthesis consists of the thermal decomposition of organometallic compounds in high‐boiling solvents containing stabilizing surfactants which regulate nucleation and growth steps. NPs obtained by thermal decomposition are well‐dispersed in organic solvents due to the hydrophobic moieties of the surfactants that stabilize the surface; thus, it is necessary to transfer them into an aqueous phase before their use in physiological environments.^62^, ^63^, ^65^, ^70^, ^83^

### 3.3 IONPs coatings and applications

As previously mentioned, once synthesized, naked NPs need a coating that prevents their aggregation, improves their colloidal stability in physiological conditions, biocompatibility, and exposes their functional groups useful for the subsequent functionalization.^84^ The strategies adopted for this purpose can be generally based on two main forces: electrostatic and steric repulsion, which can occur during or after NPs synthesis.^71^ Polymer coatings are the main class of coatings typically applied, and in particular, among them, there are sugar polymers, such as dextran and chitosan, which are the most widely used.^85^ Other polymers, such as polyethylene glycol (PEG) or polyvinylpyrrolidone (PVP), are also used, taking advantage of their hydrophilic and biocompatibility properties,^86^ particularly in the polyol synthesis method for the obtainment of IONPs.^87^ This short section will briefly discuss some of the sugar polymers used for IONPs coating.

#### 3.3.1 Dextran

Dextran is the most commonly used polymer for IONPs coating due to its high biocompatibility. It is a water‐soluble polysaccharide composed of glucose with α‐D‐(1 → 6) and α‐D‐(1 → 3) linked units, which interact with hydroxyl groups exposed by NPs.^88^ The coating procedure can be performed by adding a dextran solution to the co‐precipitation reaction^88^ or after the synthesis by heating the NPs with a dextran solution.^54^, ^58^ A further example is provided by Hradil et al., who synthesized dextran‐coated SPIONs comparing both synthesis approaches^89^; or more recently by Chen et al., who used the co‐precipitation method.^90^

#### 3.3.2 Chitosan

Chitosan is an unbranched, glucose‐based biopolymer obtained through alkaline deacetylation of chitin, which is the main component of the exoskeletons of crustaceans and insects, and is also present in some bacteria and fungi. Its positive charge, derived from the exposed amino groups, makes it easy to adhere to the surface of IONPs, interacting with hydroxyl groups.^91^ As a prime example, Khmara et al. developed a nanosystem composed of chitosan‐coated SPIONs synthesized using the co‐precipitation method and tested as a negative contrast agent in MRI applications.^92^ These types of nanosystems can also be used in the therapeutic field, for instance, as drug delivery systems,^93^ combining again both therapy and diagnostics.

#### 3.3.3 Alginate

Alginates are natural polysaccharides and linear polymers isolated from brown seaweed (*Phaeophyceae*), composed of blocks of (1,4)‐linked β‐d‐mannuronate (M) and (1‐4)‐linked β‐d‐guluronate (G) residues, and are negatively charged due to the exposure of carboxylic groups. The application of alginates is widespread in drug delivery, as excipients, in cosmetics, and others.^94^ An example of the synthesis of IONPs by co‐precipitation with a subsequent alginate‐coating is provided by Castellò et al.'s work, in which chitosan‐ and alginate‐coated IONPs are synthesized and studied in their properties of magnetism, stability and the effectiveness of the coatings for biomedical applications.^95^

### 3.4 Clinically approved and ongoing clinical trials of IONPs

IONPs in ferrofluid form were officially commercially widespread for industrial applications around 50 years ago, but in the biomedical field (e.g., for their use in magnetic hyperthermia, drug delivery, as contrast agents in MRI, etc.), they are mainly used as colloidal suspensions of superparamagnetic IONPs, and requires the approval from the Food and Drug Administration (FDA) and the European Union (EU) for their use in clinics and diagnostics. Some of them have been approved over the years and are now commercially available, while others are still in preclinical and clinical trials, as summarized in Table 1.^96^, ^97^ This short section will briefly cite some of the most relevant IONPs that are (or were in the past) approved and in trials.

**Table 1:** A summary of some IONPs.

Brand name	Generic name	Size (nm)	Surface coating	Indication	Clinical status
Feridex IV®	Ferumoxide	80–150	Dextran	Liver tumor detection	Discontinued
Endorem®	Ferumoxide	80–150	Dextran	Liver tumor detection	Discontinued
Resovist®	Ferucarbotran	60	Carboxydextran	Liver tumor detection	Discontinued Available only in Japan
Feraheme®	Ferumoxytol	30	Polyglucose sorbitol carboxymethylether	Iron‐deficiency anemia	FDA approval
Rienso®	Ferumoxytol	30	Polyglucose sorbitol carboxymethylether	Iron‐deficiency anemia	Discontinued
Sienna + ®	Magnecarbodex	60	Dextran	Breast cancer detection	EMA approval
NanoTherm®	Aminosilane‐coated SPIO	10–15	Aminosilane	Thermal ablation of GBM	FDA approval

Abbreviations: EMA, European Medicines Agency; FDA, Food and Drug Administration; GBM, glioblastoma multiforme; IONPs, iron oxide nanoparticles.

#### 3.4.1 Feridex IV®/Endorem®

Ferumoxides (Feridex IV® in the USA, Endorem® in the EU) represent the first class of IONPs dextran‐coated approved in the early 2000s for the imaging of liver lesions and for cell labeling, but were discontinued in 2008.^60^, ^97^

#### 3.4.2 Resovist®

Ferucarbotran (Resovist®) was one of the first contrast agents based on SPIONs coated with carboxydextran, approved by European Medicines Evaluation Agency (EMEA) in 2001 but discontinued in 2009 for human use, and is mainly used for liver lesions imaging and beyond.^60^ Its use in fMRI for T1 and T2‐weighted images, and for CBV measurements,^98^, ^99^, ^100^ stems from its initial diffusion. Ferucarbotran suspension, consisting of iron oxides (28 mg Fe/mL or 0.5 M) coated with carboxydextran in a hydrodynamic size of 57 nm is available only in Japan (Meito Sangyo Co., Ltd., Nagoya Research Lab.) for experimental (or preclinical) studies.^101^, ^102^ This nanosystem represents the perfect example of the multiple applications of IONPs, as it is used as a theranostic agent. In this case, it combines both its use as an MRI contrast agent (for diagnosis) and its use as a therapeutic agent, for example, in magnetic hyperthermia for cancer treatment.^103^

#### 3.4.3 Feraheme®

Ferumoxytol (Feraheme®) was approved in 2009 by the FDA for the treatment of iron deficiency anemia in patients with chronic kidney disease.^104^ This product is composed of an iron polyglucose sorbitol carboxymethylether colloid, and several institutes are producing and testing it for use in MRI as a blood pool contrast agent with clinical trials at various stages ongoing for this purpose,^60^, ^104^ which was its primary role when it was originally produced for its effectiveness in shortening T1 and T2 relaxation times.^104^ In Europe, ferumoxytol was diffused with the commercial name Rienso®, but its human use authorisation was withdrawn in 2015.

#### 3.4.4 Sienna+®

Sienna+® is a SPIONs suspension used as a tracer that follows the lymphatic system for the study of breast cancer and is coupled with a hand‐held magnetometer (Sentimag®). It was designed as an alternative to the radioactive tracer Technetium‐99m (^99m^Tc), used as a tracer for breast cancer, alone or in combination with a blue dye.^105^, ^106^, ^107^ It is currently undergoing clinical trials.

#### 3.4.5 NanoTherm®

NanoTherm® is a suspension of IONPs coated with aminosilane. It has been approved by the FDA for ablating glioblastoma multiforme (GBM) with magnetic hyperthermia treatment, and currently is undergoing clinical trials in United States for treating intermediate‐risk prostate cancer.^57^, ^97^

## 4 RECENT APPLICATIONS OF IONPS TO CBV MEASUREMENTS IN FMRI

The main goal of this chapter is to demonstrate the importance of IONPs in neuroscience research, highlighting their use in fMRI as contrast agents, and in particular, how CBV measurements taken using IONPs (specifically ferumoxytol, MION) as contrast agents are useful for a variety of brain disease research. This analysis will be carried out by presenting some recent works over the last 5 years that match the topic.

The discussion starts with an example of task‐based fMRI. In 2021, Qi et al. focused on the effects of dorsal column lesions (DC‐lesion) on the primary somatosensory cortex (area 3b) in squirrel monkeys. This lesion compromises the sense of touch in the affected hand. They studied the relationship between noninvasive fMRI measures of brain activity and invasive electrophysiological recordings with microelectrodes, before and after DC‐lesion. fMRI was essential in revealing the functional areas involved, providing a map for the subsequent high‐resolution microelectrode maps. fMRI was performed using a task‐based method, comparing both BOLD and CBV measured with MION images, and for each monkey, post‐lesion acquisitions were compared with pre‐lesion ones. Their results confirmed that the use of the hand is recovered in weeks to months and that intensive therapy of the impaired hand promotes an early recovery and cortical activation, also in almost complete lesions. fMRI was crucial for the post‐lesion reactivation studies of the primary somatosensory cortex, highlighting the neuronal activation during task‐based sessions. The comparison between BOLD and CBV MION‐mediated measurements shows that CBV scans provided stronger signals to vibrotactile digit stimulation, and that CBV has a stronger contrast‐to‐noise ratio (CNR) and covers a larger cortical area compared to BOLD profile.^108^

It is known that neuronal activation at the synaptic level of the evoked inhibitory neurons partially contributes to the hemodynamic response, and therefore to fMRI signals.^109^ However, the mechanism that brings to the hemodynamic response is unclear, and Poplawsky et al. investigated this phenomenon by observing it with fMRI while stimulating the rat olfactory bulb with pharmacological agents, which act on GABAergic granule cells.^110^ Previous fMRI studies have demonstrated that blocking N‐methyl‐d‐aspartate (NMDA) receptors using MK‐801, followed by odor stimulation, results in increased neural activity.^111^ This activity is related to an increased expression of a particular marker of neuronal activation called c‐fos mRNA, as similarly reported in other experimental contexts.^112^, ^113^ These findings suggest that NMDA receptor‐mediated synaptic interactions are critically involved in modulating the inhibitory circuit dynamics and sensory gain control within the olfactory bulb. Using CBVw fMRI acquisition during administration of NMDA receptor antagonists, Poplawsky et al. discovered a decrease in fMRI response that lasted around 30 min. MION were injected intravenously as a contrast agent for CBVw fMRI, and the acquisitions were performed before, during and after the drug administration. Specifically, they found out that the calcium influx that occurs due to NMDA receptors is involved in the synapse‐specific CBVw fMRI responses derived from the lateral olfactory tract stimulation.^110^, ^114^

**Figure 4:**
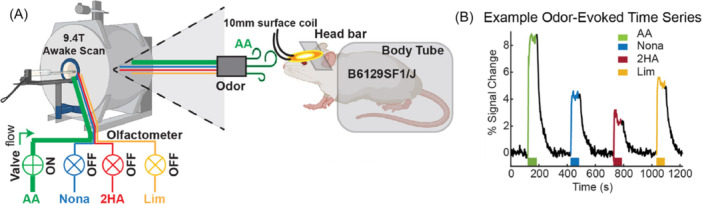
Studies using the olfactory bulb and cerebral blood volume‐weighted (CBVw) images using monocrystalline iron oxide nanocrystals (MION) as a contrast agent. (A). Poplawsky et al. performed their studies on the olfactory bulb using a modified air‐dilution olfactometer able to selectively deliver up to four odorant molecules (acetate, nonanal, 2‐hydroxyacetophenone and limonene) inside the instrument, exploiting pneumatic control valves.^115^ (B). There is an example odor‐evoked time‐series data from the glomerular layer of the bulb during awake CBVw fMRI scans.^116^ [Color figure can be viewed at wileyonlinelibrary.com]

Subsequently, in 2023, Poplawsky et al. continued their studies using the olfactory bulb and CBVw images using MION as a contrast agent, but in awake mice. This time, they demonstrated that high‐resolution fMRI can measure small activations in mice without the use of anesthesia, capturing behavior‐associated neuronal activity and spontaneous resting‐state activity. To do this, they acquired odor‐specific activation pattern maps related to four different smells at high resolution, and then examined layer‐dependent olfactory adaptation to repeated exposures between the glomerular layer and the granule cell layer (Figure 4). These patterns were found to be spatially distinct, and activations were greater in superficial layers, decreasing with laminar depth. They also proved that neuronal activity is intact in awake mice with respect to anesthetized rats (by comparing this study with their previous works), and that fMRI signals, in response to olfactory adaptation, are more attenuated in the granule cell layer with respect to the glomerular layer.^116^

Han et al., in their 2021 research, studied the striatal circuit, whose abnormalities are involved in psychiatric and neurological disorders,^117^ overcoming the two main problems with in vivo fMRI: first, the spatial resolution of the conventional 3 T fMRI is limited; secondly, fMRI does not allow a direct perturbation of the brain systems. For their purpose, they coupled electrical microstimulation with ultrahigh‐field 7 T fMRI, using MION as a contrast agent to induce CBVw contrast in two rhesus monkeys (nonhuman primates). These results were compared with their previous resting‐state acquisitions.^118^ The stimulation was performed at different points along the dorsal‐to‐ventral axis, revealing a dorsoventral functional organization both within the striatum and thalamus, and across the cortex, and how the anatomical configuration forces neuronal activation within the caudate and putamen.^117^

Working with animal models by in vivo fMRI requires the use of anesthetic drugs (e.g., urethane^119^), which impact vascular physiology.^119^ For this reason, Le et al. decided to investigate the impact of different doses of dexmedetomidine and isoflurane on CBF and CBV (thus changes in cerebral perfusion) in mice. The method consists in fluxing for 5 s nitrogen gas, generating a transient hypoxia condition to perform BOLD‐dynamic susceptibility contrast (BOLD‐DSC) measurements of the whole‐brain. They set up different experiments, comparing physiological conditions during hypoxia stimuli, optimizing the BOLD measures and their repeatability, and applying the differential concentrations of the two anaesthetic drugs. Among these experiments, they compare CBV measurements obtained by BOLD‐DSC with those obtained using MION as contrast agent, and in this case, they demonstrated that these methods lead to similar acquisition with a high voxel correlation (according to voxel‐wise Pearson's *r* correlation coefficients). They discovered that dexmedetomidine generally reduces cerebral perfusion, while isoflurane has an opposite dose‐dependent effect.^120^, ^121^

In the research carried out in 2024 by Ma et al., the response of the dorsomedial striatum (DMS) circuit and the medial orbitofrontal cortex (mOFC) following oxycodone withdrawal in rats was studied. Their work consisted in training rats to self‐administer oxycodone for 2 weeks, then inducing a voluntary abstinence for 2 weeks. They tested the effects of GABAa and GABAb receptor agonists to inactivate mOFC and DMS for the understanding of their role in opioid seeking. They combine the use of these agonists with fMRI, and in particular, they used fMRI to determine both the effect of DMS inactivation on CBV and its functional connectivity with other brain regions. CBVw images were taken using again Feraheme® as contrast agent. The results demonstrate that DMS inactivation leads to a decrease of CBV not only in DMS but also in other cortical regions and highlight the DMS's functional connections with other brain areas such as frontal, sensorimotor and auditory regions. This suggests that DMS could be a potential target for relapse prevention.^122^

**Figure 5:**
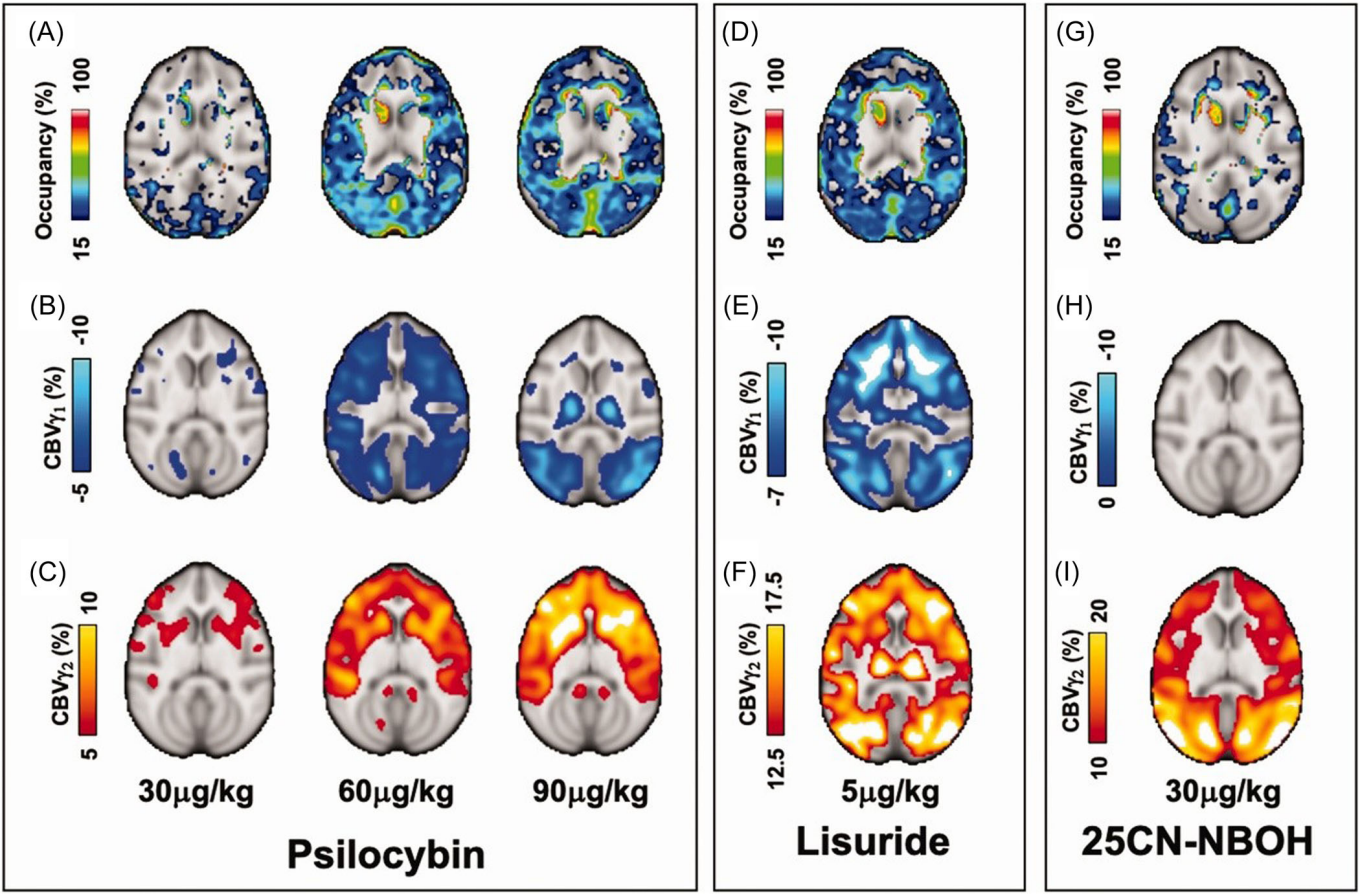
Representative axial images of dose‐dependent occupancy (%) of serotonin 2A receptor (5‐HT_2A_R) and CBV (%) maps from decreased CBV (γ_1_) and increased CBV (γ_2_) fits for psilocybin (A, B, C), lisuride (D, E, F) and 25CN‐NBOH (G, H, I).^123^ [Color figure can be viewed at wileyonlinelibrary.com]

Another phMRI‐based research, conducted by Bagdasarian et al., studies the neuromodulatory effects of some agonist drugs for serotonin 2A receptor (5‐HT_2A_R) in nonhuman primates, including psilocybin, lisuride and 25CN‐NBOH, in the treatment of mental health disorders. Particularly, these researchers combined positron emission tomography (PET), used for the measure of the receptor occupancy with the help of a radioligand for the receptor; and phMRI, used for drugs‐induced hemodynamic changes (i.e. CBV changes acquired using MION) (Figure 5). They reported that 25CN‐NBOH resulted in larger changes in CBV compared to psilocybin and lisuride, and that receptor occupancy is higher for psilocybin and lisuride (specifically, the first one has a saturation at 60 mg/kg dose) and lower for 25CN‐NBOH (linear to CBV changes).^123^

**Figure 6:**
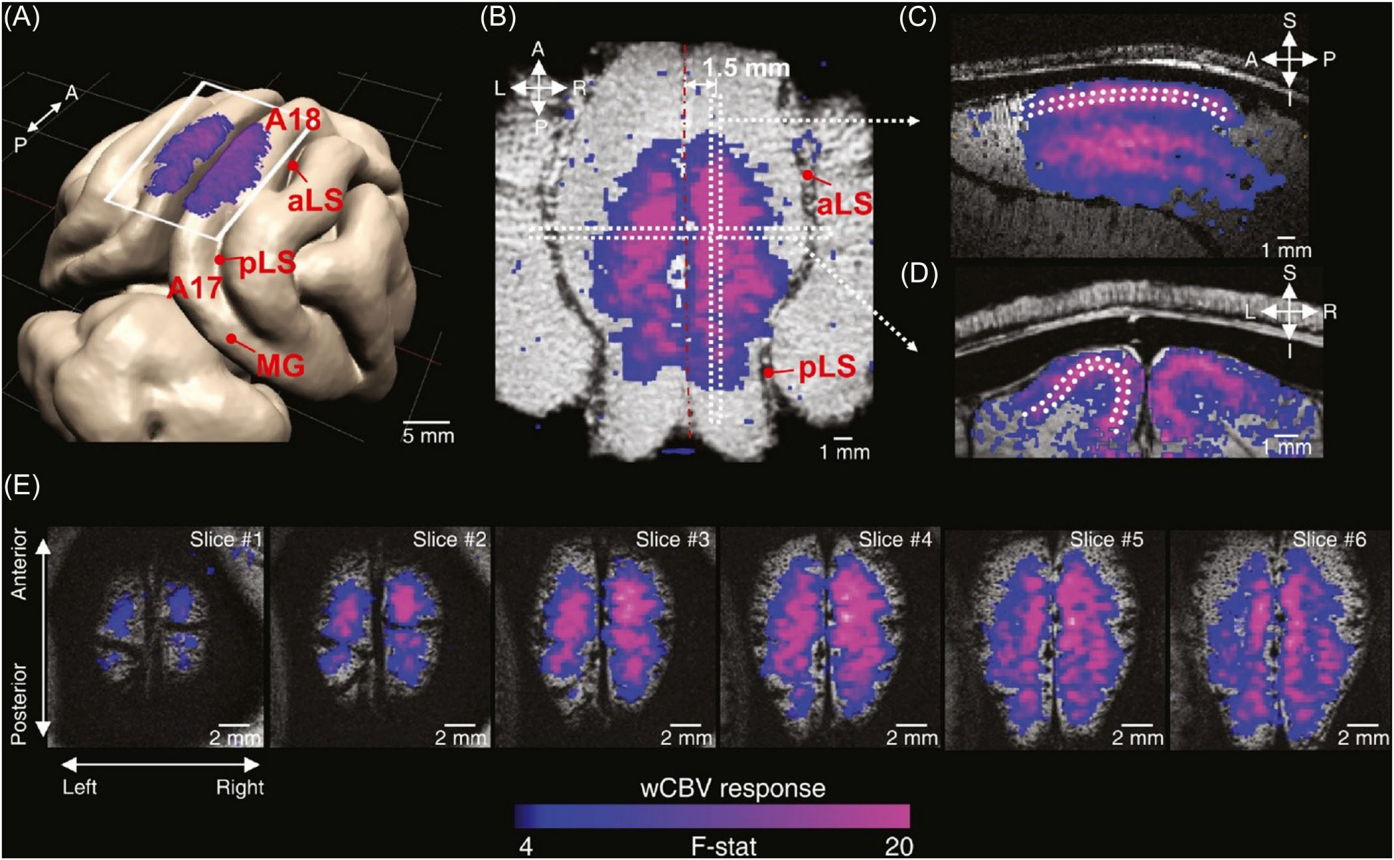
3D rendered cat brain model, with the stimulus‐evoked signal changes observed on the cortical surface with monocrystalline iron oxide nanocrystals (MION) administered as contrast agent. (A). Stimulus‐evoked cerebral blood volume weighted (wCBV) changes are observed in the axial (B), sagittal (C), and coronal (D) planes. Activation maps for six contiguous 250 μm thickness axial slices (E). The scale bar refers to the F‐statistics of the general linear model (GLM).^124^ aLS, anterior lateral sulcus; MG, marginal gyrus; pLS, posterior lateral sulcus. [Color figure can be viewed at wileyonlinelibrary.com]

An interesting study on the cat primary visual cortex (V1), carried out by Cho et al. in 2022, exploits CBVw fMRI with the aim of detecting layer‐specific orientation‐selective responses in V1. CBVw images are coupled with single‐vessel resolution two‐ and three‐photon optical imaging, useful for measuring arterial dilation and blood velocity in response to visual stimuli. Images were taken using a high‐resolution 9.4 T MR system, with an intravenous bolus of MION administered as contrast agent (Figure 6). The researchers demonstrated that coupling these imaging makes it possible to reveal a laminar response pattern that highlights systematic changes in selectivity across cortical layers of V1, which resides in neural circuitry. This is particularly evident in layer 4, compared to the other cortical layers.^124^

Recently, in 2025, Shen et al. conducted a study on the relationship between cocaine self‐administration (SA) and the increase in impulsive decision‐making in low‐impulsive rats. Their study aimed to classify rats based on their impulsivity into low‐, middle‐, and high‐impulsive groups to examine how chronic cocaine SA impacts decision‐making, associating these changes with alterations in brain functionality, particularly with dopamine (DA) receptor expression. The rats were classified by performing a delay‐discounting task (DDT) procedure for 6 weeks. CBV images were acquired by injecting Feraheme® as a contrast agent via tail vein catheter, and DA receptor mRNA expression was analysed with RNAscope in situ hybridization assay. Chronic cocaine SA resulted in increased impulsivity in low‐impulsive rats, suggesting that cocaine could be a factor involved in developing impulse behavior in normally nonimpulsive subjects. These results were correlated with resting‐state fMRI analysis, which highlighted a positive correlation between impulsivity and regional CBV in the midbrain, thalamus, and auditory cortex in low‐impulsive rats. A related reduction in DA receptor expression was also found.^125^

## 5 CONCLUSIONS

The main application of CBVw fMRI using IONPs is related to animal studies. As emerged from the previously discussed works, IONPs play a crucial role in enhancing the sensitivity of the fMRI method by increasing CNR with respect to other acquisition modalities in T2‐weighted images, and shortening the T2 and T2* relaxation times. These factors highlight the potential power of coupling CBV measurements with the appropriate contrast agent in fMRI studies, enabling a deeper understanding of brain functions under experimental conditions. The use of this method in neuroscience has a wide range of applications, ranging from the study of neuronal disorders or the brain after physical lesions to behavioral and pharmacological studies.

The versatility of IONPs in terms of applications, the diversity of synthesis methods, and the possibility for coatings and functionalization make them an ideal theranostic agents, which combine therapeutic effects with clinical diagnosis. However, not all of the IONPs mentioned before are currently approved for human use, particularly as blood pool agents, some of which are still undergoing clinical trials. Their approval for human use by the FDA is mainly limited to their therapeutic role, as seen for Feraheme® and NanoTherm® suspensions.

The hope for the future is that the formulations currently in clinical trials will be approved, allowing the full potential of IONPs to be realized in the biomedical field. This would enable their use to enhance early disease diagnosis, improve the understanding of brain functions that are currently difficult to study or unknown, and support therapy‐coupled diagnosis.

## AUTHOR CONTRIBUTIONS

Giorgio Capuzzello drafted, revised, and edited the manuscript. Antonella Antonelli and Rosaria Rinaldi also contributed by writing, revising, and editing the manuscript. Riccardo Di Corato developed the concept, and both wrote, revised, and edited the manuscript. All authors reviewed and approved the final version of the manuscript.

## CONFLICT OF INTEREST STATEMENT

The authors declare no conflicts of interest.

## ETHICS STATEMENT

Not applicable.

## Data Availability

Not applicable as no new data are generated in this study.
